# Distributed interactive brain circuits for object-in-place memory: A place for time?

**DOI:** 10.1177/2398212820933471

**Published:** 2020-06-30

**Authors:** John P. Aggleton, Andrew J.D. Nelson

**Affiliations:** School of Psychology, Cardiff University, Cardiff, Wales, UK

**Keywords:** Anterior thalamus, hippocampus, perirhinal cortex, entorhinal cortex, nucleus reuniens, recency, recognition, retrosplenial cortex, thalamus, space

## Abstract

Rodents will spontaneously learn the location of an individual object, an ability captured by the object-in-place test. This review considers the network of structures supporting this behavioural test, as well as some potential confounds that may affect interpretation. A hierarchical approach is adopted, as we first consider those brain regions necessary for two simpler, ‘precursor’ tests (object recognition and object location). It is evident that performing the object-in-place test requires an array of areas additional to those required for object recognition or object location. These additional areas include the rodent medial prefrontal cortex and two thalamic nuclei (nucleus reuniens and the medial dorsal nucleus), both densely interconnected with prefrontal areas. Consequently, despite the need for object and location information to be integrated for the object-in-place test, for example, via the hippocampus, other contributions are necessary. These contributions stem from how object-in-place is a test of associative recognition, as none of the individual elements in the test phase are novel. Parallels between the structures required for object-in-place and for recency discriminations, along with a re-examination of the demands of the object-in-place test, signal the integration of temporal information within what is usually regarded as a spatial-object test.

## Introduction

When I walk into my office and notice that specific items have been re-arranged, I am immediately curious. It is not something that I can simply ignore, my attention being drawn to those items that are now in the ‘wrong’ place. In just the same way, the attention and exploration of rodents are drawn to familiar objects whose locations within an arena have moved since the animal last explored the apparatus. This behaviour occurs because the animal had previously learnt both the ‘what’ and the ‘where’ of the objects in its environment. This spontaneous form of learning is captured by the ‘object-in-place’ (OiP) test (Dix and Aggleton, 1999; see also Poucet et al., 1986), making it a valuable tool for the study of memory mechanisms. One reason for its popularity is that the linking of a specific item (object) with a particular location (place) appears to capture key aspects of episodic learning. All that is missing is the temporal component to complete the what? where? and when? of animal episodic-like memory (Aggleton and Pearce, 2001; Dere et al., 2007; Iordanova et al., 2008; see also Eacott and Easton, 2010).

This review begins by describing the OiP test, preceded by two closely related behavioural tests that might be regarded as ‘precursors’. These two tests are ‘spontaneous object recognition (OR)’ and ‘spontaneous object location (OL) recognition’. If these tests are truly precursors, then any lesion that disrupts OR or OL recognition will also disrupt OiP. A further prediction is that some brain sites required for OiP will not be necessary for either of the two ‘precursor’ tests. This second prediction presumes that additional cognitive processing is needed to bring these two elements together in an effective manner.

For sites within the temporal lobe, testing these predictions can be set within an anatomical framework that assumes a partial degree of segregation of ‘what’ and ‘where’ information prior to reaching the hippocampus (Aggleton, 2012; Diana et al., 2007; Ranganath and Ritchey, 2012). Beyond the temporal lobe, further candidate sites are considered, typically based on one or both of two characteristics. First, the site is closely connected with medial temporal structures. Second, the site has been implicated in human memory disorders, most especially anterograde amnesia. Initial information is drawn from lesion studies, but further information comes from experiments that assess the levels of neuronal activity associated with experiencing either item (object) novelty or spatial novelty in intact rodent brains, for example, via immediate-early gene (IEG) expression. The focus is largely on c-*fos* activity given its importance for OR (Seoane et al., 2012).

### Behavioural considerations

#### OR

**Figure 1.**
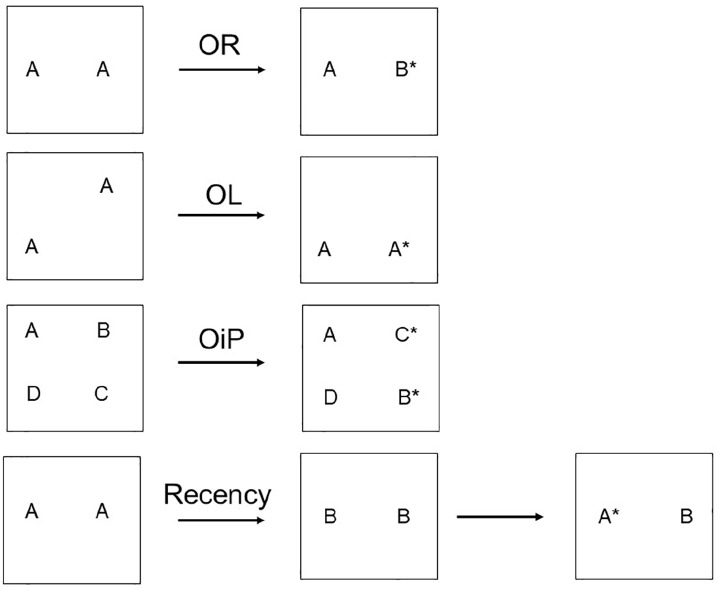
Schematic of testing protocols for object recognition (OR), object location (OL), OiP, and object recency. The different letters correspond to different objects. The asterisks mark those objects in the test trial typically preferred by a normal rodent as they have a novel element or, in the case of recency, are the furthest back in time.

Following its introduction by Ennaceur and Delacour (1988), the spontaneous OR test has been adopted worldwide, providing a standard assay of nonspatial learning. The test consists of two phases. In the sample phase, the rodent is typically allowed to explore two duplicates of novel object (A, A) in a familiar space, for example, a walled arena (Figure 1). In the test phase, the rodent is reintroduced to the arena where it can explore freely between the now-familiar object (A) and a novel object (B). Because the objects occupy the same locations in the arena as those used in the sample phase, location cues should be redundant. Variants on the basic design have been devised for the Y-maze (McTighe et al., 2010) and for continuous-trial testing (Albasser et al., 2010a; Ameen-Ali et al., 2012).

While the OR test is often regarded as nonspatial, this characterisation may be misleading. We know from OiP that rodents spontaneously associate identity with location. It is, therefore, possible that object novelty information is supplemented by the confirmatory knowledge that this particular object did not previously occupy this location (see Chao et al., 2016a). The benefit of such additional information might, however, be difficult to determine experimentally as the magnitude of OR discrimination can be poor at reflecting memory strength or persistence (Cole et al., 2019). Finally, OR is often described as a test of familiarity discrimination to underline how it is more than just object identification (but see Bussey and Saksida, 2007).

#### OL recognition

Using a similar logic, Ennaceur et al. (1997) looked at the preference rats might show for exploring a familiar object that has been moved to a new location within an arena (see also Poucet et al., 1986). In a typical version of the spontaneous OL test, two identical objects (A, A) are placed apart in an arena for the sample phase, for example, each in separate corners (Figure 1). For the test phase, one of the identical objects is moved to a previously unoccupied corner (A, A), the expectation being that normal rodents will preferentially explore the familiar item in a new location. It is important to remember that the animal does not have to learn the identity of the object, merely where something was previously located. Nevertheless, the appreciation that both objects remain the same will affect overall exploration and may, therefore, impact on the indices of novel location discrimination, as measured behaviourally.

#### OiP

In its original version (Dix and Aggleton, 1999; see also Poucet et al., 1986), the rat freely explores a square arena containing four different objects (A, B, C, and D), each one close to a different corner (Figure 1). Following this sample phase, the rat is removed. Next, two of the objects (B, C) are re-arranged so that they swap corner positions while the other two objects (A, D) remain in the same location (Figure 1). Normal rats typically then spend more time exploring the two moved objects (B, C), reflecting their ability to learn individual object–location pairings. A subsequent variant on this design (Eacott and Norman, 2004; Langston and Wood, 2010) starts with just two different objects in the sample phase (A, B), but then exposes the animal to two identical, familiar objects in the test phase in the same locations (A, A). Now, one of the two ‘A’ objects is in a location not previously occupied by that object and so should be preferentially explored. While OiP requires location learning, it makes fewer demands on navigation skills than tasks such as the Morris water maze. This difference stems from how the goals of the rodent’s exploration (objects) remain highly visible throughout OiP.

#### Other considerations

All three tests involve ‘spontaneous’ learning. For this reason, it is important to examine the total amount of object exploration during the sample phase of any study. These data help determine whether spontaneous levels of initial exploration are affected by the experimental manipulation, a potential confound (Kinnavane et al., 2015). This is a complex issue as a deficit in the mnemonic process being studied might itself affect total exploration times during this same sample phase. To take the case of an animal with an OR memory deficit, it could be predicted that objects in the sample phase might, as a default, be treated as novel (if the animal has no memory of previous items). Conversely, sample objects might be treated as familiar (if the animal has false memories of previous experiences), resulting in decreased levels of sample exploration. In practice, the large majority of studies involving rats with perirhinal cortex lesions report normal sample exploration behaviour, despite deficits in subsequent OR tests (Aggleton, 2018; Albasser et al., 2015; Olarte-Sánchez et al., 2015; but see McTighe et al., 2010).

A further issue is that performance of spontaneous tests is prone to considerable variability. This variability comes from a number of sources, including inter-animal differences, the choice of objects, and the subjective nature of the behavioural scoring. Consequences include the difficulty of measuring the severity of any deficit and how best to interpret null results. Solutions include increasing the sample size in each group and obtaining data from more repeat trials, for example, by adopting continuous tests of recognition that provide multiple trials per session (Albasser et al., 2010a; Ameen-Ali et al., 2012).

### Brain sites for spontaneous OR

Several reviews have considered the impact of lesions and related manipulations on spontaneous OR (Brown et al., 2010; Dere et al., 2007; Ennaceur, 2010; Warburton and Brown, 2010, 2015; Winters et al., 2008). For this reason, the following section, which largely focusses on studies of rats, only provides an overview.

Beginning with Ennaceur et al. (1996; see also Mumby and Pinel, 1994), there is almost uniform agreement that the perirhinal cortex is required for effective spontaneous OR (Brown et al., 2010). Perirhinal lesions repeatedly lead to a lack of difference in the test phase between the times spent with the novel object and the familiar object (e.g. Albasser et al., 2011, 2015; Barker et al., 2007; Bussey et al., 1999; Mumby et al., 2007; Olarte-Sánchez et al., 2015; Winters et al., 2004). Furthermore, the greater the loss of perirhinal cortex tissue, the greater the recognition deficit (Albasser et al., 2009). There is also evidence that the deficit becomes more robust as the length of the interval between sample and test is increased (Cole et al., 2020; Norman and Eacott, 2004) and when the objects contain overlapping features (Bussey et al., 2002; Norman and Eacott, 2004). As visual information reaches the perirhinal cortex from area Te, it is perhaps unsurprising that removal of this area can also disrupt OR (Ho et al., 2011). Linked with this result is the finding that perirhinal lesions do not impair rat OR in the dark (Albasser et al., 2011), that is, this is principally a visual recognition deficit.

In contrast, the impact of hippocampal lesions on OR remains contentious. Numerous studies report seemingly normal OR performance after extensive hippocampal lesions, even after lengthy retention intervals (e.g. Ainge et al., 2006; Barker and Warburton, 2011b; Forwood et al., 2005; Good et al., 2007; Langston and Wood, 2010; Mumby, 2001; Mumby et al., 2002; Winters et al., 2004; see also Aggleton et al., 1986; Cole et al., 2019). At the same time, in what appears to be a smaller number of studies, OR deficits are found after hippocampal damage (e.g. Broadbent et al., 2010; Clark et al., 2000; for reviews see Barker and Warburton, 2011b; Mumby, 2001). Post hoc attempts to reconcile these discrepant findings that, for example, there is a hidden spatial component in those OR studies associated with a hippocampal deficit, have yet to reach an agreement. A related, more tractable, suggestion is that hippocampal lesions disrupt object exploration rather than recognition per se (Ainge et al., 2006), resulting in an apparently inconsistent picture. Perhaps the only safe conclusion is that OR can often be performed effectively despite hippocampal lesions.

The entorhinal cortex is of interest because it provides a reciprocal link between the perirhinal cortex and hippocampus. As the lateral entorhinal cortex is particularly interconnected with the perirhinal cortex (Doan et al., 2019; Naber et al., 1997), it might be anticipated that this entorhinal division is potentially the more important for OR memory. In fact, lateral entorhinal lesions often spare OR (Kesner et al., 2001; Kuruvilla and Ainge, 2017; Wilson et al., 2013a, 2013b; see also Parron and Save, 2004), although deficits have been reported (Hunsaker et al., 2013). The implication is that an intact perirhinal cortex is typically sufficient for OR, suggesting that lateral entorhinal cortex lesions only become effective under restricted conditions. One possible example is when OR involves local spatial features (Kuruvilla and Ainge, 2017).

Meanwhile, the medial entorhinal cortex is more interconnected with the postrhinal cortex, rather than the perirhinal cortex (Naber et al., 1997). Consistent with this connectivity, neither lesions of the postrhinal cortex (Norman and Eacott, 2005) nor the medial entorhinal cortex (Hunsaker et al., 2013; Kesner et al., 2001; Kuruvilla and Ainge, 2017; Parron and Save, 2004) appear to disrupt OR.

Beyond the temporal lobe, it has so far proved impossible to find lesion sites consistently associated with OR deficits in rodents. The long list of sites that have been examined includes the mammillary bodies (Nelson and Vann, 2014, 2017), the anterior thalamic nuclei (Dumont and Aggleton, 2013; Mitchell and Dalrymple-Alford, 2005; Moran and Dalrymple-Alford, 2003; Warburton and Aggleton, 1999; Wilton et al., 2001), the medial dorsal thalamic nucleus (Cross et al., 2013; Mitchell and Dalrymple-Alford, 2005), intralaminar thalamic nuclei (Mitchell and Dalrymple-Alford, 2005), nucleus reuniens (Barker and Warburton, 2018), the medial prefrontal cortex (mPFc), including prelimbic cortex (Ennaceur et al., 1997; Hannesson et al., 2004a; Mitchell and Laiacona, 1998), the anterior cingulate cortex (Ennaceur et al., 1997), and the retrosplenial cortex (Ennaceur et al., 1997; Hindley et al., 2014; Parron and Save, 2004; Vann and Aggleton, 2002; but see De Landeta et al., 2020). Of these many null results, the apparent lack of effect of lesions in the anterior and medial thalamic nuclei is notable as recognition memory deficits are typically seen in human diencephalic amnesia (Aggleton et al., 2011) as well as in monkeys with medial thalamic lesions tested on delayed nonmatching-to-sample (DNMS) (Aggleton and Mishkin, 1983). While there are important differences between spontaneous OR tests for rodents and tests of recognition given to humans and monkeys, studies with monkeys suggest that spontaneous tests are more sensitive than DNMS (Nemanic et al., 2004; Pascalis et al., 2004), implying that the null results in rats are not due to test insensitivity.

The initial conclusion is, therefore, that selective parts of the parahippocampal region, most notably perirhinal cortex, and their sensory inputs are both necessary and sufficient for rodent OR. Consistent with this conclusion, the cutting of major tracts linked to the temporal lobe, such as the cingulum bundle (Ennaceur et al., 1997) or the fornix (Ennaceur et al., 1996, 1997; Easton et al., 2009; Warburton and Aggleton, 1999) does not affect standard OR tests.

This same conclusion is supported by studies of IEG expression (Aggleton et al., 2012). When rats are passively shown novel visual images, there is a consistent rise in c-*fos* expression in both the perirhinal cortex and visual area Te (Aggleton et al., 2012; Wan et al., 1999; Zhu et al., 1995, 1996). In contrast, other sites, including the hippocampus, postrhinal cortex, and entorhinal cortex, fail to show similar c-*fos* responses (Aggleton et al., 2012). Likewise, no sites beyond the temporal lobe show reliable IEG activity responses to novel stimuli (Barbosa and Silva, 2018).

When rats receive multiple OR trials in which they can actively explore the objects, for example, in a bow-tie shaped maze, the pattern of c-*fos* expression changes. While increase in perirhinal cortex and area Te c-*fos* expression is again seen, they are now accompanied by activity changes in the hippocampus and entorhinal cortex (Albasser et al., 2010b; Kinnavane et al., 2016). One possible explanation for this difference is that the active exploration of objects at opposite ends of the maze not only engages spontaneous object learning but also spontaneous OL learning.

### Brain sites for spontaneous OL memory

As might be expected, the pattern of OL deficits following selective lesions is markedly different from that seen for OR. This difference reflects the emphasis on spatial rather than object-based information. One simple prediction is that temporal lobe sites needed for allocentric spatial learning will be required for this task. Consistent with this prediction, hippocampal lesions are associated with OL deficits (Barker and Warburton, 2011b; Mumby et al., 2002; Save et al., 1992), while perirhinal cortex lesions spare performance (Barker et al., 2007), consistent with the spatial:nonspatial double dissociation between these two sites (Aggleton et al., 1997; Bussey et al., 1999; Chao et al., 2016b; Winters et al., 2004). Furthermore, transient disruptions of dorsal hippocampal activity and plasticity are sufficient to impair OL (De Landeta et al., 2020; Migues et al., 2019; Yamada et al., 2017; Yu et al., 2018), though the same manipulations spare OR (De Landeta et al., 2020; Yamada et al., 2017; Yu et al., 2018). Finally, lesions of the fornix, one of the major tracts of the hippocampus, can also impair OL performance (Ennaceur et al., 1997; Warburton et al., 2000), although the deficit may be mild (Bussey et al., 2000).

In view of the importance of the hippocampus, it might be expected that entorhinal cortex lesions will also impair OL. Such deficits have been found following large entorhinal lesions involving both its medial and lateral divisions (Parron and Save, 2004). Meanwhile, lesions of the lateral entorhinal cortex appear to be without effect (Wilson et al., 2013a, 2013b), a result consistent with the lack of effect of perirhinal lesions on this task. Furthermore, crossed disconnections of the lateral entorhinal cortex and mPFc are also without effect (Chao et al., 2017). These null results point to the potential significance of the medial entorhinal cortex for this test.

Beyond the temporal lobe, there is less evidence of cortical sites required for OL memory. Two exceptions, however, are the retrosplenial cortex and parietal cortex, where lesions impair the reaction to the location change in an object when identity is not important (Parron and Save, 2004; Save et al., 1992). Furthermore, extensive lesions combining both the anterior cingulate and retrosplenial cortices also impair OL (Ennaceur et al., 1997). In addition, transient retrosplenial lesions can disrupt OL learning (De Landeta et al., 2020).

Meanwhile, a number of diencephalic sites are required for allocentric processing, most notably the anterior thalamic nuclei and the mammillary bodies, yet remain to be directly tested on OL. Current evidence shows that crossed lesions of the anterior thalamic nuclei and fornix are sufficient to impair OL (Okada and Okaichi, 2006; Warburton et al., 2000), implicating these thalamic nuclei. In contrast, cingulum bundle lesions may spare OL (Ennaceur et al., 1997) despite the many retrosplenial and anterior thalamic fibres in this pathway.

Finally, studies examining the contributions of the mPFc have repeatedly shown that this area is not needed for OL performance (Barker et al., 2007; Cross et al., 2013; Ennaceur et al., 1997; Poucet, 1989). Likewise, the anterior cingulate area is not required (Ennaceur et al., 1997). Lesions of the medial dorsal thalamic nucleus, a defining thalamic relay for prefrontal cortex, also spare OL (Cross et al., 2013). Similarly, lesions of nucleus reuniens, which despite being directly connected with both the mPFc and hippocampus, do not affect OL performance (Barker and Warburton, 2018).

### Brain sites for spontaneous OiP

The simplest prediction is that sites required for either OR or OL are also required for OiP (Table 1). This ‘precursor’ prediction is clearly supported by the deficits seen on OiP tests following lesions of the perirhinal cortex (Barker et al., 2007; Barker and Warburton, 2008, 2015; but see Eacott and Norman, 2004) and hippocampus (Barker et al., 2017; Barker and Warburton, 2011b; Warburton and Brown, 2010), as well as crossed unilateral lesions in these two sites (Barker and Warburton, 2015). While perirhinal cortex seemingly provides object-based information, the hippocampus is presumably required for the allocentric placement of individual objects (Albasser et al., 2013; Chao et al., 2016b; Langston and Wood, 2010) and their integration (Diana et al., 2007). Consistent with this account, lesions of the lateral entorhinal cortex can also impair OiP (Wilson et al., 2013b), although postrhinal cortex lesions may spare performance (Norman and Eacott, 2004). The lateral entorhinal cortex lesion effects presumably reflect its substantial perirhinal inputs, alongside its less dense postrhinal inputs (Doan et al., 2019).

**Table 1.** Pattern of behavioural findings following lesions in various targets sites following assessment with spontaneous object recognition (OR), object location (OL), object-in-place (OiP), and temporal discrimination (recency) tests.

Brain site	OR	OL	OiP	Recency
Area Te	X			
Perirhinal cortex	X	√	X	X
Postrhinal cortex	√		√	
Entorhinal cortex	√ X	√	X	√*
Hippocampus	√ X	X	X	X
Fornix	√	X	√ X	
Retrosplenial cortex	√	X	X	√ X
Anterior cingulate cortex	√	√		
Anterior thalamic nuclei	√	X*	X	√ X
Mammillary bodies/MTT	√		X	√ X
Medial prefrontal cortex	√	√	X	X
Medial dorsal thalamic N	√	√	X	X
Nucleus reuniens	√	√	X	
Cingulum bundle	√	√		

OR: object recognition; OL: object location; MTT: mammillothalamic tract.

Findings for the lateral and medial entorhinal cortex are combined.

See text for relevant references.

√: unimpaired; √ X: less severe or inconsistent deficits; √*: crossed lateral entorhinal and medial prefrontal lesions (Chao et al., 2016a); X:: impaired; X*: crossed unilateral lesion with contralateral fornix lesion (Okada and Okaichi, 2006; Warburton et al., 2000).

Parallel findings relating to many of these same temporal areas come from IEG activation studies. When familiar visual items are spatially reconfigured and viewed, akin to OiP, c-*fos* expression changes are now seen in the hippocampus and subiculum, but not in the perirhinal cortex or area Te (Wan et al., 1999). This pattern is the opposite of that seen for visual item novelty (Wan et al., 1999). Likewise, performance of a radial-arm maze working memory task that involved the rearrangement of familiar spatial cues led to selective c-*fos* expression changes in hippocampal fields (CA1, CA3, and dentate gyrus) as well as the postsubiculum, but not in the perirhinal cortex, medial or lateral entorhinal cortex, or postrhinal cortex (Jenkins et al., 2004).

There is a concern is that the spatial demands of the OL test are not as exacting as those for OiP. Consider the most frequent version of the OiP test, where two objects are moved (interchanged) and two remain in the same location for the test phase. The result is that the configuration of *all four* objects has changed; that is, the relative positions of all neighbouring items for every object is different from that in the sample phase (Figure 1). Unless the animal appreciates absolute location, aided by an accurate sense of direction, it will struggle to detect which two objects have interchanged position. For this reason, OiP performance may be particularly sensitive to factors such as the height of the arena walls, whether the walls are uniform, the salience of the distal spatial room cues, and even how the animal is introduced into the arena (Langston and Wood, 2010). Meanwhile, the OL test may require less spatial resolution as it often involves the novel presence of an object in one quadrant of an arena that had previously been unoccupied. For these reasons, other brain sites required for allocentric memory, such as the anterior thalamic nuclei (Mitchell and Dalrymple-Alford, 2006; Sutherland and Rodriguez, 1989; Warburton et al., 1999; Wolff et al., 2008), the mammillary bodies (Sziklas and Petrides, 1998; Vann and Aggleton, 2003), and the retrosplenial cortex (Lukoyanov et al., 2005; Vann and Aggleton, 2002) should all prove critical for OiP, even if their importance for OL is less consistent. Matching this prediction, lesions of the anterior thalamic and lateral dorsal nucleus (Wilton et al., 2001), the mammillothalamic tract (Nelson and Vann, 2014), and retrosplenial cortex (Vann and Aggleton, 2002) all impair OiP.

A further factor to consider is that OiP assesses ‘associative recognition’. This term refers to how the objects or locations involved in the OiP test phase are individually familiar, but their *combination* is novel. (In OR and OL, the individual object or individual location is novel.) Consequently, the next question is whether additional sites are required for OiP, that is, more than just the sum of those needed for the two ‘precursor’ tests such as OR and OL (Table 1).

One such additional site is the mPFc. Lesions in this area consistently block OiP performance but spare both OR and OL (Barker et al., 2007; Barker and Warburton, 2015; Cross et al., 2013; Warburton and Brown, 2015). Furthermore, the disruption of dopamine signalling in the mPFc impairs the encoding, but not retrieval, stages of OiP (Savalli et al., 2015). Meanwhile, disconnection studies show that the mPFc functions in close cooperation with both the perirhinal cortex (Barker and Warburton, 2015) and hippocampus (Barker et al., 2017; Barker and Warburton, 2015; Warburton and Brown, 2010) to support OiP. The finding that fornix lesions have inconsistent effects on OiP (Bussey et al., 2000; Eacott and Norman, 2004) suggests that other routes linking the hippocampus with the mPFc, or vice versa, may contribute. One such route is via lateral entorhinal cortex, as suggested by a disconnection analysis (Chao et al., 2016a).

Another frontal-hippocampal route is via nucleus reuniens in the thalamus (Herkenham, 1978; Varela et al., 2014). While the mPFc does not directly innervate the hippocampus, it does reach the hippocampus via a monosynaptic link involving nucleus reuniens (Prasad and Chudasama, 2013). Functional support for the contribution of this indirect route comes from evidence that lesions of nucleus reuniens impair OiP when testing longer retention delays (Barker and Warburton, 2018). At the same time, lesions of the medial dorsal thalamic nucleus, which is densely and reciprocally connected with mPFc, also impair OiP (Cross et al., 2013). A similar OiP deficit is seen after crossed unilateral lesions between the mPFc and the medial dorsal nucleus (Cross et al., 2013), confirming the importance of their interaction.

### What is special about the OiP task?

**Figure 2.**
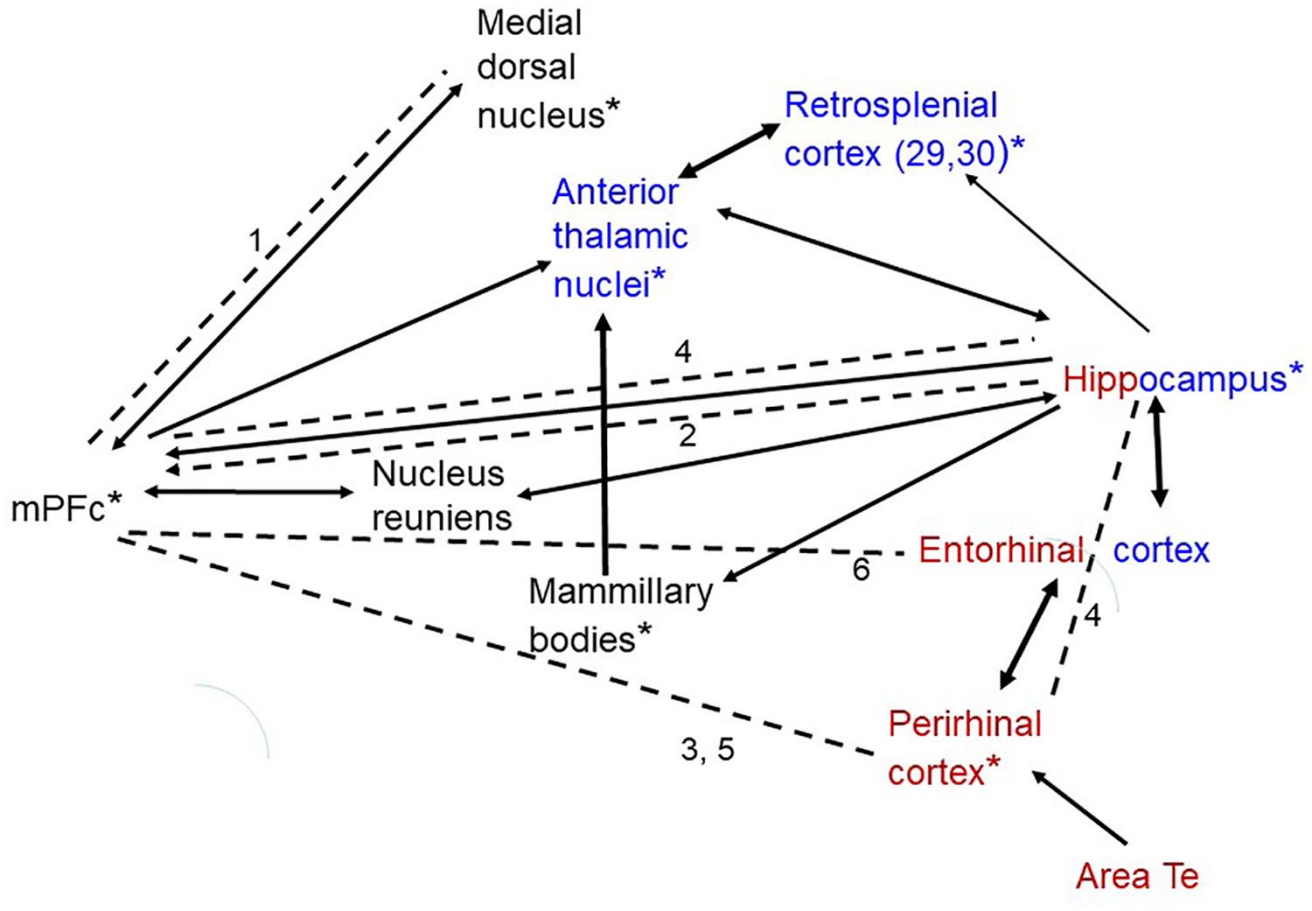
Network of structures supporting OiP. All structures indicated are required for performance of OiP. The black arrows indicate major interconnecting pathways. The thicker arrows represent particularly dense projections. Red – structures also required for object recognition. Blue – structures also required for object location. *Structures required for spontaneous recency discriminations (for nucleus reuniens and area Te, it is not yet known if required for recency). Dashed lines indicate structures that function together to support OiP, as shown by disconnection (arrows if known direction of effect). 1: Cross et al. (2013); 2: Barker et al. (2017); 3: Barker and Warburton (2008); 4: Barker and Warburton (2015); 5: Barker et al. (2007); 6: Chao et al. (2016a). mPFc: medial prefrontal cortex; MTT: mammillothalamic tract.

It is clear from Table 1 that performing the OiP task relies on a far more complex, distributed network than either of its two precursor tasks, OR and OL (Figure 2). This realisation raises important questions about the nature of associative recognition and why, for example, the mPFc is so vital. From the outset, it is important to appreciate that mPFc lesions often spare spatial memory tasks (e.g. De Bruin et al., 1994, 2001; Hannesson et al., 2004b; Joel et al., 1997), despite their consistent importance for OiP, that is, the deficit is unlikely to be a failure of allocentric learning. This distinction is even more convincing when considering the medial dorsal thalamic nucleus. While lesion of this nucleus disrupt OiP (Cross et al., 2013), they repeatedly have little or no effect on spatial memory tasks (Hunt and Aggleton, 1991; Kolb et al., 1982; Mitchell and Dalrymple-Alford, 2005), highlighting how the OiP deficit after lesions of the mPFc and its key connections is not spatial per se, rather it involves additional processes.

Before examining potential, additional processes, it is necessary to consider whether the emergence of these extra sites simply reflects an increase in test difficulty, leaving the OiP test more sensitive than either OR or OL. This account is, however, difficult to sustain given the high performance levels of control animals on OiP, contrasting with chance levels by animals with medial prefrontal interventions (e.g. Barker et al., 2007; Barker and Warburton, 2008). A more specific concern is that, as already noted, the spatial demands of OiP might sometimes be greater than those for OL. Furthermore, if OiP is seen as the conjunction of OL and OR then, by combining these processes, the test becomes more prone to error, that is, more difficult, than either OL or OR. These descriptions will not, however, explain the emergence of sites, such as the medial dorsal thalamic nucleus, not required for either OL or OR (Cross et al., 2013; see Table 1). The implication is that additional processing is required for the effective integration of these different information types. Simply calling this ‘task difficulty’ hides the nature of these extra demands.

One clue comes from the striking parallel between brain sites required for OiP and those required for object recency discriminations (Table 1, recency). Recency discriminations (Figure 1) test the spontaneous ability of rodents to select between familiar objects that differ with regard to the times since they were last experienced, with normal rats preferring to explore objects from further back in time (Mitchell and Laiacona, 1998). One valuable step has been to show that the preference for the object furthest back in time is not simply because that item has effectively been forgotten and, hence, treated as if novel. This explanation can be discounted by the dissociations between impaired recency discriminations and intact familiarity judgements for matched stimuli (Albasser et al., 2012; Barker et al., 2019; Fortin et al., 2002).

Sites required for tests of object recency not only include the mPFc (Hannesson et al., 2004a; Mitchell and Laiacona, 1998) and the hippocampus (Albasser et al., 2012; Barker and Warburton, 2011b; see also Kesner et al., 2010) but also the medial dorsal thalamic nucleus (Cross et al., 2013; Mitchell and Dalrymple-Alford, 2005) and perirhinal cortex (Barker and Warburton, 2011a; Hannesson et al., 2004a; Warburton and Brown, 2010). Results from related spontaneous tests of stimulus recency also implicate the anterior thalamic nuclei (Dumont and Aggleton, 2013; Wolff et al., 2006), the mammillothalamic tract (Nelson and Vann, 2017), and retrosplenial cortex (Powell et al., 2017). While there is evidence that the recency deficit found after anterior thalamic, mammillothalamic tract, and retrosplenial cortex damage is not as profound as that observed after either medial prefrontal or hippocampal lesions, c-*fos* activity levels in retrosplenial cortex correlate with object recency performance (Powell et al., 2017).

Perhaps the most intuitive account of object recency is that it reflects relative differences in trace strength since experiencing the two objects from differing times in the past (Ennaceur, 2010; Marshuetz and Smith, 2006). A more formal version, based on the relative strengths of memory traces, has been derived from the sometimes opponent processes (SOP) model in which stimuli, when experienced, pass through a series of activation states in a serial order (Wagner, 1981). Following an initial primary state associated with stimulus attraction and exploration, there follows a secondary state, associated with weaker approach behaviour. This is followed by a final, inactive stimulus state. The preference for older items in recency tests reflects the preferential exploration of items in this final state over those items still in the secondary state (Tam et al., 2014, 2015) This preference occurs because the final state stimulus can immediately return to an ‘attractive’ primary state. A strength of this explanation is that it is embedded within a highly influential model of associative learning. A weakness is the post hoc nature of deciding the current ‘state’ of a stimulus. For example, rats can distinguish between two objects experienced 1 hour apart, after a subsequent delay of 24 h (Mitchell and Laiacona, 1998), implying that between 24 and 25 h, the objects change ‘state’. At the same time, many other studies use much shorter retention intervals to test recency effectively.

To gain a better understanding of the processes involved in recency discrimination, trace strength models were examined systematically by varying the numbers of items in the list and the inter-stimulus intervals, prior to subsequent recency testing (Barker et al., 2019). There was, however, a repeated failure to show that the length of the interval between two sequential objects predicted levels of subsequent recency discrimination (Barker et al., 2019), as expected by trace strength models and by SOP. Instead, there may be multiple processes that can guide temporal order choice (Marshuetz and Smith, 2006). These processes include the greater overlap between neural representations of stimuli closer together in time (Manns et al., 2007), relative trace strength discrimination (Ennaceur, 2010), the chaining of item–item associations, including sequential episodes (Marshuetz, 2005), and the appreciation of the time elapsed since salient events (Roberts et al., 2008). While this same variety of potential mechanisms (Marshuetz, 2005) may help to explain the range of brain sites that appear to contribute to recency judgements (Table 1), the same logic could be turned on in its head to predict partial sparing as the various mechanisms might compensate each other.

A different approach to the parallel recency and OiP results is to consider the task demands. In OiP, the animal separates and compares the sample phase from the test phase in a way that is not required in the OR and OL tests, where one item (OR) or one location (OL) is novel and, thus, automatically draws attention. In OiP, no individual object or location is novel; consequently, it is necessary to contrast the sample and test phases, that is, maintain their temporal distinction and reduce interference. This ability relates to the concept of identifying on ‘which’ occasion an event occurred (Eacott and Easton, 2010) as part of the challenge of distinguishing overlapping spatial information. For these same reasons, the impact of hippocampal lesions on OiP may be on both the spatial and temporal aspects of the test (Barker et al., 2017). A similar combination of spatial and temporal deficits could potentially exacerbate the OiP deficits following lesions of the anterior thalamic nuclei, mammillary bodies, and retrosplenial cortex.

This temporal discrimination role closely relates to the notion that the mPFc acts back upon the hippocampus to reduce interference between memory representations (Eichenbaum, 2017). For example, mPFc lesions impair temporal judgements between arms in a radial maze but not judgements of spatial novelty in the same maze, that is, it is not an underlying spatial deficit (Hannesson et al., 2004b). As already noted, one potential pathway for this temporal function is via nucleus reuniens, which preferentially projects to the ventral hippocampus (Prasad and Chudasama, 2013). Consistent with this interpretation, it is evident that these same temporal judgements are more reliant on the ventral, rather than dorsal, hippocampus (Howland et al., 2008).

The present analysis also highlights how few sites in the rodent brain are critical for OR (Table 1). One consequence is that despite the use of OR in countless behavioural experiments, it can provide a very limited behavioural screen for nonspatial learning. At the same time, the OiP test is clearly sensitive to dysfunction in a much wider neural network, in which multiple processes coordinate. Consequently, using both OiP and OR creates a superior behavioural screen. While it may be more difficult to pinpoint the underlying cause of an OiP impairment, the same test helps to combat the false negatives that will arise from relying on the standard OR test.

A goal of this review is to derive a network of structures that support OiP. Relevant information comes not only from the impact of lesions in one site (Table 1) but also from disconnection studies, as well as IEG activity measures in normal rodent brains (Aggleton et al., 2012; Barbosa and Silva, 2018). Figure 2 provides a preliminary attempt to combine these various sources of information. It is inevitable that such a framework will be simplistic. To take one example, the supposed distinction between spatial versus object-based information within the medial and lateral entorhinal cortices, respectively, has been challenged, leading to a more nuanced position (Doan et al., 2019; Knierim et al., 2014). For this reason, these two subareas are combined in Figure 2.

Taken together, several messages arise from this analysis. The most obvious concerns the step change in functional demands from when the rodent has to detect and respond to novelty (object or spatial) compared to when responding to the novelty formed by new combinations of familiar stimuli (‘associative recognition’). In the latter situation, the mPFc becomes critical, seemingly irrespective of the type of associative recognition task. Furthermore, the mPFc appears to support multiple functions integral to OiP. For this same reason, OiP brings into play other areas that function closely, but in different ways, with the mPFc to ensure its effectiveness (e.g. the medial dorsal thalamic nucleus and nucleus reuniens), while the ability to communicate with distal sites, including the hippocampus, also appears to rely on thalamic sites, for example, nucleus reuniens and the anterior thalamic nuclei (Prasad and Chudasama, 2013). Given that projections from the hippocampus to the mPFc may also be integral to OiP (Barker et al., 2017), this inter-relationship appears to be reciprocal. It is anticipated that advances in procedures that allow the manipulation of individual pathways, for example, via optogenetics, will bring new insights into this functional network (Figure 2), revealing its undoubted true complexity.

A theme throughout this review has been the need to consider carefully the behavioural demands of these spontaneous tests and not simply see them as direct measures of recognition memory, location memory, or the combination of these two. Key issues include the importance of considering the sample exploration data as well as the test phase data (Ainge et al., 2006). As has been discussed, there might be reasons to suppose that a deficit in the process being investigated might, by its very nature, alter sample exploration. At the same time, differences in sample exploration can confound interpretation. Other issues concern the ways in which exploration data are extracted across the length of the test phase in order to ensure that the most appropriate information is used (Dix and Aggleton, 1999). Further issues centre on how object exploration is best defined and measured, including whether to analyse total exploration times, total bouts of exploration, or both (Olarte-Sánchez et al., 2015). Added complexities arise from the potential impact of stress and arousal on performance (Roozendaal et al., 2018). It is evident that the apparent simplicity of these spontaneous tests should not mask their underlying complexity. Finally, a case is made that OiP contains temporal elements, which by adding to the ‘what’ and ‘where’ elements of the test, brings its demands closer to episodic-like memory problems.
